# Editorial

**Published:** 1841-01-09

**Authors:** 


THE MEDICAL EXAMINER.

PHILADELPHIA, JANUARY 9, 1841.

### INSANE POOR.

Our readers are aware that within a few years past the number of asylums for the insane poor has been gradually increasing, and that each state has felt the necessity of providing a place of refuge for the insane who are past recovery, and of treatment for those whose disease is still curable. The county almshouses, or the general hospitals designed for the treatment of medical and surgical diseases, are notoriously unfitted for the treatment of lunatics. That is, for the treatment of those whose condition requires the adoption of moral means, and of soothing influences, which can only be secured in an asylum specially devoted to the insane, and furnished with the necessary space, and means of amusement and relaxation.

In the state of Pennsylvania, it is very probable that an asylum will soon be founded for the reception of the insane poor of the commonwealth; the necessity of it is generally admitted, and nothing but the pecuniary difficulties of the state can prevent the speedy endowment of the asylum. We do not, however, think that this delay is much to be regretted; our error is hasty, rather than slow legislative action,—and if a project of this nature be acted upon, without due deliberation, it will probably be found that the buildings are not the best adapted for the purpose, or that the rules regulating the admission of patients were defective.

The latter point is one of the most difficult to determine. Who are to be regarded as insane? This is a point full of difficulties, not merely with reference to the mental ability or disability of the patients, but whether their mental disorder is of a nature to require or justify admission into an hospital.

When the asylums are open indiscriminately to all who are deprived of their reason, the number of inmates is constantly increasing, and a strong temptation is offered to members of families to relieve themselves of the burthen of an idiotic or even imbecile member, and to support him at the charge of the public; hence, the natural effect of institutions which admit patients with little restriction, is to increase the apparent number of the insane. By restricting those entitled to admission to such as are either troublesome or dangerous to society at large, and to those whose case is still susceptible of cure, the inconvenience of public institutions ceases, and they produce decidedly beneficial results.

An institution conducted upon this restricted footing, is not attended with any great expense, and is positively required by the necessities of civilization; and we do not doubt that the different states will be provided with asylums which are large enough for the probable numbers of the insane,—for it is plainly required by ordinary principles of humanity to provide special institutions for the treatment of the insane, who require conditions which cannot be afforded by general hospitals.

---

We shall shortly commence the publication of a series of extracts from Dr. Meigs' obstetric case-book, classified and commented upon by the author. Upon the value of contributions of this character from such a source, it is not necessary to dwell, feeling satisfied as we do, that we could offer nothing more acceptable to our readers.

